# Syndrome de Kartagener de découverte fortuite au cours d'un bilan d'infécondité du couple à propos d'un cas

**DOI:** 10.11604/pamj.2019.33.316.16919

**Published:** 2019-08-21

**Authors:** Amadou Doumbia, Youssouf Koné, Abdoulaye Koné, Oumou Maïga, Adama Dembélé

**Affiliations:** 1 Service de Radiologie du Centre de Santé de Référence de la Commune VI, Bamako, Mali; 2 Service de Radiologie du Centre Hospitalier Universitaire Régional de Brest, Brest, France; 3 Service de Radiologie du Centre Hospitalier Jacques Boutard, Saint-Yrieix-la-Perche, France; 4 Service de Radiologie du CHU du Point G, Bamako, Mali; 5 Service d'Imagerie Médicale du CHU Gabriel Touré, Bamako, Mali; 6 Service de Radiologie du CHU de Yopougon, Abidjan, Côte d'Ivoire

**Keywords:** Kartagener, stérilité, imagerie, Kartagener, sterility, imaging

## Abstract

Le syndrome de Kartagener est une entité particulière parmi les dyskinésies ciliaires primitives (DCP) caractérisée par une triade clinique: sinusite, bronchectasie et situs inversus complet ou incomplet. C'est une maladie congénitale rare à transmission autosomique récessive. Nous rapportons un cas de syndrome de Kartagener dans un contexte d'infertilité avec une akinospermie au spermogramme.

## Abstract (en)

Kartagener's syndrome is a rare primitive ciliary dyskinesia (DCP) characterized by a clinical triad: sinusitis, bronchiectasis and complete or incomplete situs inversus. It is a rare congenital autosomal recessive disease. We report a case of Kartagener syndrome in an infertile couple with akinospermia detected using spermogram.

## Introduction

Le syndrome de Kartagener est une entité particulière parmi les dyskinésies ciliaires primitives (DCP) caractérisée par une triade clinique sinusite, bronchectasie et situs inversus complet ou incomplete [1]. C'est une maladie congénitale rare à transmission autosomique récessive qui survient chez environ une personne sur 15000 [2]. Les infections des voies aériennes au cours de la pathologie se manifestent classiquement dès l'enfance [2]. La revue de la littérature africaine nous a permis de retrouver des études publiées sur cette pathologie en Afrique du nord [3, 4]. Le syndrome de Kartagener en plus des infections des voies aériennes supérieures et pulmonaires peut entrainer la stérilité chez l'homme [5]. L'infertilité au cours du syndrome de Kartagener est la conséquence d'une diminution ou une immobilité des spermatozoïdes secondaire à une atteinte ultrastructurale de leurs flagelles, identique à celle observée au niveau des cils respiratoires. Nous rapportons un cas de cette pathologie rare, révélée par une infertilité primaire en abordant l'apport de l'imagerie dans le diagnostic et revue de la littérature.

## Patient et observation

Homme de 35 ans qui consulte avec son épouse pour une infécondité primaire depuis 4 ans. Le patient est tabagique actif (15 paquets/année) avec des antécédents d'épisodes d'obstruction nasale, de rhinorrhée chronique à répétition sur fond de toux et de dyspnée asthmatiforme depuis l'enfance. Son épouse n'a aucun antécédent particulier et les explorations (bilan hormonal et radiologique) réalisées dans le cadre de l'infertilité du couple étaient normales. L'examen clinique du patient était normal en dehors de discrètes râles crépitants à l'auscultation au niveau des bases pulmonaires. Il n'y avait pas d'anomalie des organes génitaux externes. Au spermogramme, on mettait en évidence une akinétospermie avec numération normale des spermatozoïdes. La radiographie pulmonaire objectivait une dextrocardie sans foyer infectieux pleuropulmonaire. L'échographie prostatique et testiculaire étaient normales. Une polypose naso-sinusienne était retrouvée au scanner des sinus de la face. Devant ces résultats, un syndrome de Kartagener fut évoqué, d'où la réalisation d'un scanner thoraco-abdominopelvien et une biopsie ORL. Le scanner thoraco-abdominopelvien confirme le situs inversus complet associé à une bronchectasie. L'histologie de la biopsie ORL retrouve une anomalie structurelle des cils vibratiles. L'enquête familiale n'a pas retrouvé de consanguinité ou de cas similaire dans la fratrie.

## Discussion

**Figure 1:**
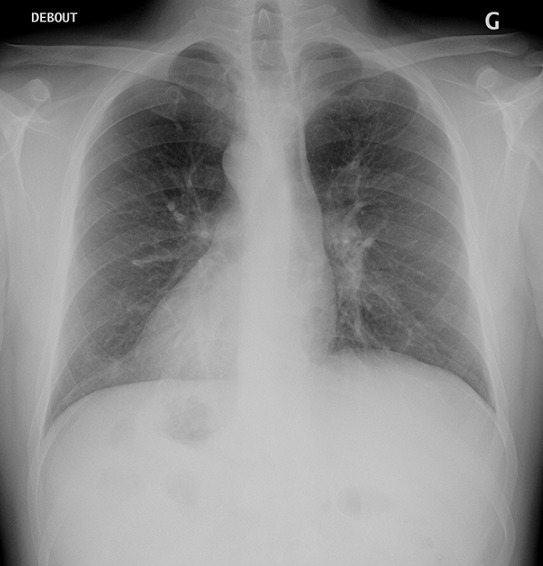
Radiographie thoracique de face: dextrocardie, bouton aortique droit, inversion avec une position en miroir de l'opacité hépatique à gauche et de la poche à air gastrique à droite

**Figure 2:**
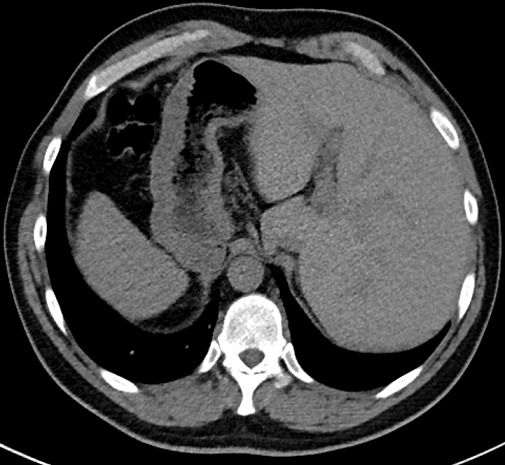
Scanner abdominal, coupe axiale en fenêtre parenchymateuse mettant en évidence à droite la rate et l'estomac, le foie est localisé dans l'hypochondre gauche

**Figure 3:**
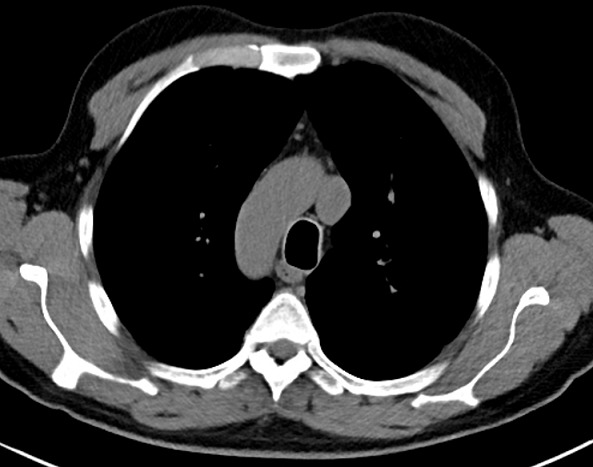
Scanner thoracique, coupe axiale en fenêtre médiastinale visualisant une crosse aortique orientée à droite

**Figure 4:**
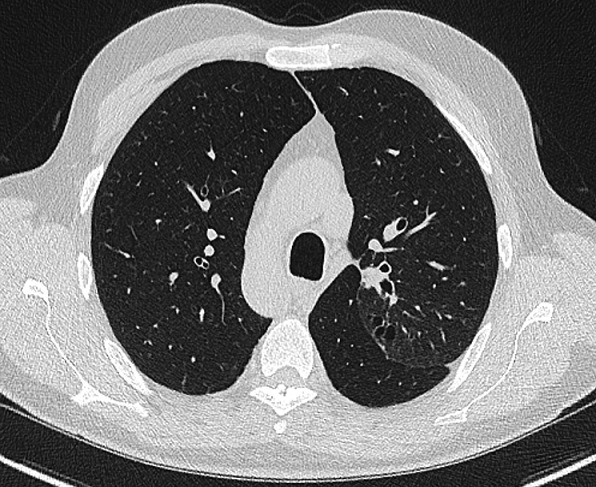
Scanner thoracique, coupe axiale en fenêtre parenchymateuse retrouvant une bronchectasie bilatérale avec quelques impactions mucoïdes

**Figure 5:**
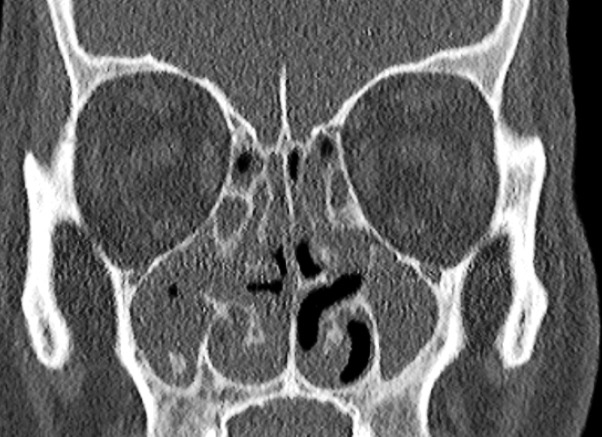
Scanner des sinus de la face en reconstruction coronale objectivant un comblement avec épaississement polypoïde de la muqueuse des sinus de la face

Décrit pour la première fois par Manes Kartagener en 1935, le syndrome qui porte son nom associe une sinusite chronique, un situs inversus et une bronchectasie [6]. Le syndrome de Kartagener représente 50% des dyskinésies ciliaires primitives (DCP). C'est une maladie génétique rare, à transmission autosomique récessive; cependant des modes de transmission lié à X ou dominant ont été également décrits [6]. Il s'agit d'une mutation de gènes codant pour la dynéine au niveau des chromosomes 5, 9 et 7 responsables d'une anomalie morphologique et/ou fonctionnelle des cils [6]. L'âge de diagnostic de l'affection varie selon les auteurs avec une révélation de la maladie dès l'enfance [3, 4, 6, 7]. Dans notre observation le patient était un homme de 35 ans, similaire au cas de Prisca Gabrielle *et al.* [1] qui rapportaient un cas chez l'adulte. La prédominance masculine est retrouvée par Moreau [6]; par contre Melki [4] rapportait une série de 6 patientes. Certains auteurs ont rapporté des cas en période néonatale [3]. Dans notre cas, l'enquête n'a retrouvé aucun antécédent familial de maladie respiratoire chronique et de consanguinité chez les parents. L'imagerie joue un rôle central dans le bilan de la pathologie. Ainsi dans notre cas, la dextrocardie était retrouvée à la radiographie standard et l'échographie cardiaque (Figure 1). Le scanner thoraco-abdominal (Figure 2, Figure 3, Figure 4) a montré un situs inversus complet (dextrocardie, localisation droite de la rate, de l'estomac et gauche du foie et de la vésicule biliaire). Aussi l'anomalie de rotation concernait les poumons et les vaisseaux abdominaux avec la veine cave inférieure à gauche de l'aorte). En fenêtre parenchymateuse pulmonaire, on retrouvait au scanner une discrète bronchectasie cylindrique du lobe moyen, de la lingula et des lobes supérieurs. L'atteinte bronchique concerne le plus souvent le lobe moyen et le lobe inférieur avec majoration des bronchectasies avec l'âge [1]. La symptomatologie du syndrome de Kartagener est dominée par les signes respiratoires débutant dès l'enfance. Ils sont présents chez tous les patients et ne sont spécifiques que par leur chronicité et leur récurrence annuelle [1]. Il s'agit d'un encombrement bronchique chronique, d'une toux grasse quotidienne avec des sécrétions muco-purulentes et des phases d'exacerbations et de surinfections. Dans notre observation, le patient présentait un tableau bronchopneumopathie obstructive avec bronchectasies diffuses comme décrit dans la littérature [6] avec une toux chronique asthmatiforme glaireuse sans dyspnée. L'évolution de la pathologie respiratoire est variable, elle dépend notamment de la précocité du diagnostic et de la rigueur de la prise en charge [2]. L'atteinte ORL notamment des voies aériennes supérieures est marquée par des sinusites et des otites en rapport avec l'anomalie de l'épuration mucociliaire. Dans notre cas, le patient a présenté des épisodes d'obstruction nasale à répétition et une rhinorrhée chronique récidivante évoluant depuis l'enfance. Au scanner des sinus de la face, on retrouvait un comblement avec épaississement muqueux polypoïde pan sinusien évocateur d'une polypose naso-sinusienne (Figure 5). La polypose naso-sinusienne est présente chez environ 30% des patients. Plusieurs facteurs sont impliqués dans leur survenue (facteurs génétiques, immunologiques, intolérance à l'acide acétyl-salicylique). Ils se voient dans plusieurs maladies inflammatoires naso-sinusiennes et sont l'expression non spécifique de la maladie muqueuse sous-jacente [3, 4, 6].

La particularité de notre observation réside dans le faite que le syndrome de Kartagener est découverte lors d'un bilan de stérilité. Ce mode de découverte du syndrome de dyskinésie ciliaire primitive est peu décrit dans la littérature [5]. La stérilité est souvent associée aux autres signes de la maladie comme dans notre cas. Quatre vingt dix à 100% des patients atteints de dyskinésie ciliaire primitive sont stériles [6]. Mais selon Ceccaldi *et al.* [5], la fréquence de la stérilité chez les patients atteints de dyskinésie ciliaire primitive est probablement moins importante, la plupart des études ayant été faites dans des centres spécialisés en stérilité. Notre patient avait consulté pour une infertilité primaire après 4 ans de vie de couple et présentait sur deux spermogrammes successifs une akinétospermie sans anomalie de la numération des spermatozoïdes. L'infertilité est due à une hypo ou une immotilité des spermatozoïdes, secondaire à une atteinte ultra structurale de leurs flagelles, en général identique à celle observée au niveau des cils respiratoires. Le nombre de spermatozoïdes est normal au spermogramme; on parle donc de stérilité par asthénospermie. En dehors de leur motilité diminuée, les spermatozoïdes sont potentiellement fécondants. Contrairement à la mucoviscidose, les voies spermatiques et notamment les épididymes ne sont pas atteints dans la dyskinésie ciliaire primitive [3, 5]. Des publications montrent que les patients souffrant du syndrome de Kartagener avec des anomalies ultra structurales ciliaires typiques ont toutefois une motilité normale des spermatozoïdes [2, 5]. D'autres auteurs [2, 3, 5 ,6] proposent d'examiner l'ultra structure des cils bronchiques chez tout homme stérile en présence d'une anomalie de la gaine fibreuse du flagelle. Il pourrait s'agir, dans ces cas, d'une forme mineure de dyskinésie ciliaire primitive ne se manifestant que par une stérilité [6]. La prise en charge de la stérilité masculine liée au syndrome de Kartagener fait appel à la fécondation in vitro par la réalisation d'un test de vitalité des spermatozoïdes (HOST: Hypo Osmotic Swelling test) suivie d'une micro injection des ovocytes [5]. Quant à la prise en charge globale du syndrome de Kartagener, il repose essentiellement sur la kinésithérapie respiratoire de drainage bronchique, l'antibiothérapie en cas de surinfection, une couverture vaccinale adéquate anti grippale et anti pneumococcique [3-6].

## Conclusion

La stérilité masculine est un mode de découverte du syndrome de Kartagener comme dans notre observation et doit être suspecté en présence d'une akinospermie lors du spermogramme surtout en l'absence d'anomalie du bilan de la conjointe.

## Conflits d’intérêts

Les auteurs ne déclarent aucun conflit d'interêts.
